# "Attending to History" in Major System Change in Healthcare in England: Specialist Cancer Surgery Service Reconfiguration

**DOI:** 10.34172/ijhpm.2022.6389

**Published:** 2022-03-14

**Authors:** Catherine Perry, Ruth J. Boaden, Georgia B. Black, Caroline S. Clarke, Sarah Darley, Angus I.G. Ramsay, David C. Shackley, Cecilia Vindrola-Padros, Naomi J. Fulop

**Affiliations:** ^1^Applied Research Collaboration Greater Manchester, Division of Population Health, Health Services Research and Primary Care, University of Manchester, Manchester, UK.; ^2^Division of Nursing, Midwifery & Social Work, School of Health Sciences, Faculty of Biology, Medicine & Health, University of Manchester, Manchester, UK.; ^3^Alliance Manchester Business School, University of Manchester, Manchester, UK.; ^4^Department of Applied Health Research, University College London (UCL), London, UK.; ^5^UCL Research Department of Primary Care and Population Health, University College London (UCL), London, UK.; ^6^Centre for Primary Care and Health Services Research, University of Manchester, Manchester, UK.; ^7^Christie NHS Foundation Trust, Manchester, UK.; ^8^Institute of Cancer Sciences, Manchester Academic Health Science Centre (MAHSC), Manchester, UK.; ^9^Department of Targeted Intervention, University College London (UCL), London, UK.

**Keywords:** Major System Change, Reconfiguration of Services, Centralisation, History, Qualitative Research

## Abstract

**Background:** The reconfiguration of specialist hospital services, with service provision concentrated in a reduced number of sites, is one example of major system change (MSC) for which there is evidence of improved patient outcomes. This paper explores the reconfiguration of specialist oesophago-gastric (OG) cancer surgery services in a large urban area of England (Greater Manchester, GM), with a focus on the role of history in this change process and how reconfiguration was achieved after previous failed attempts.

**Methods:** This study draws on qualitative research from a mixed-methods evaluation of the reconfiguration of specialist cancer surgery services in GM. Forty-six interviews with relevant stakeholders were carried out, along with ~160 hours of observations at meetings and the acquisition of ~300 pertinent documents. Thematic analysis using deductive and inductive approaches was undertaken, guided by a framework of ‘simple rules’ for MSC.

**Results:** Through an awareness of, and attention to, history, leaders developed a change process which took into account previous unsuccessful reconfiguration attempts, enabling them to reduce the impact of potentially challenging issues. Interviewees described attending to issues involving competition between provider sites, change leadership, engagement with stakeholders, and the need for a process of change resilient to challenge.

**Conclusion:** Recognition of, and response to, history, using a range of perspectives, enabled this reconfiguration. Particularly important was the way in which history influenced and informed other aspects of the change process and the influence of stakeholder power. This study provides further learning about MSC and the need for a range of perspectives to enable understanding. It shows how learning from history can be used to enable successful change.

## Background

**Key Messages**

** Implications for policy makers**

- “Attending to history” can be influential in major system change (MSC) and consideration of the power of stakeholders is important at all stages of the process.
- History is multifaceted and will be interpreted differently by stakeholders. The power dynamics of history may require specific consideration.
- Maintaining an awareness of how any previous change attempts have affected the willingness of local stakeholders to engage in change is important.

** Implications for the public**

Changes to the way in which health services are delivered can be necessary to ensure that everybody gets the best care possible, and resources are used in the most effective way. This study examines how, by being knowledgeable and aware of the history of previous attempts to change the way health services are provided, those organising changes can make them happen. An important part of understanding history is the views and experiences of the public to whom services are delivered, as well the views of the various professionals and organisations. This study encourages those proposing and undertaking change to take account of these stakeholder views, and to understand what happened in the past and why, which should help ensure that services are changed to be acceptable to everyone involved.

Major system change (MSC) in healthcare is defined as “coordinated, system-wide change affecting multiple organisations and care providers, with the goal of making significant improvements in efficiency of healthcare delivery, the quality of patient care, and population-level patient outcomes”^1^ (p. 422). The reconfiguration of specialist hospital services, with service provision concentrated in a reduced number of sites, is one example of MSC for which there is evidence of improved patient outcomes in some contexts.^2^ However, little is known about the processes through which reconfiguration of services can be achieved and there is an increasing call for research to draw on a range of perspectives to increase understanding of MSC.^3^

 A realist review of the literature on MSC in healthcare identified a framework of five ‘simple rules’ for enhancing implementation: combine designated and distributed leadership; establish feedback loops; attend to history; engage physicians; and involve patients and families.^1^ The authors argued that analysis of history is important in implementing MSC, although it is not predictive of how change might or might not happen. This review appeared to take a “History-as-Fact” perspective on history, where “*past decisions influence present decisions*”^4^ (p. 21), as suggested by some path-dependent models of change.^5^ Whilst the authors of the review “sought to avoid a deterministic view of history,”^1^ (p. 439) where history may not predict the future, there appeared to be little consideration of different perspectives on history.

 The availability of personal and documentary historical accounts and awareness of, and interest in, the history of change by change leaders, are important in shaping how history can be ‘attended to.’^6^ Previous work has shown that these accounts may influence the process of MSC in several ways: educating change leaders about previous change attempts and their outcomes; enabling problematic situations to be avoided or better managed^1,7^; and enabling leaders to build on familiar and valued ideas and activities, possibly replicating previous successes.^6^ Leaders of the centralisation of specialist cancer surgery in London used examples of successful MSC to demonstrate that change was possible.^8^ However Best et al^7^ caution that technology, ideology and environment may change between episodes of change, restricting the utility of past MSC to inform future change. May et al^9^ have emphasised the importance of context and argue that much evaluative work seeks to eliminate contextual confounders, rather than viewing context as the state into which change must be integrated.

 Although other authors have discussed ‘building on what already exists,’^10^ the attend to history rule has not been greatly expanded upon since being described by Best et al.^1^ One study that did so,^11^ concluded that multiple stakeholder voices within change processes could thwart leaders’ ability to transform services in response to historical evidence. The aim of this paper is to explore the reconfiguration of specialist oesophago-gastric (OG) cancer surgery services in a large urban area of England, with a focus on attending to history. This focus is chosen as it was evident early on in data analysis that the history of change attempts was important to those involved in planning and implementing the changes studied in this research.

 In our discussion we draw on the work of Suddaby and Foster,^4^ who outlined four models or perspectives on history which exist on a continuum between an objective and a subjective view (History-as-Fact, History-as-Power, History-as-Sensemaking and History-as-Rhetoric). We demonstrate how history was not only perceived as “fact” in our study, but also as “power” and “sensemaking.” This also leads to the identification of the interactions between the rules, and some limitations of the simple rules framework. The study extends existing knowledge about how history may influence other aspects of change processes, and particularly the importance of stakeholder power within that.

## Methods

 This study draws on qualitative research (observations/documentary evidence/interviews) from a mixed-methods evaluation of the reconfiguration of specialist cancer surgery services in Greater Manchester (GM).^8^ The data is drawn from a larger study which included services in London, with this paper focusing on data from GM only.The mixed-methods evaluation commenced in September 2015 and involved analysis of data from both London and Manchester. However, by the end of 2015 OG cancer surgery in London had been reconfigured, which enabled a study of implementation and outcomes using both qualitative and quantitative data and has been reported elsewhere.^12^ Changes in GM did not take place until late 2018 so outcomes were not captured, but a contemporaneous study of how change was planned, enabled, and moved forward was possible that was not captured so clearly in the London data.

###  Background and Setting

 There are longstanding recommendations to reconfigure OG specialist cancer surgery services in order to reduce variations in access,^13^ increase patient volumes and improve outcomes (high volume is associated with better outcomes).^14,15^ In GM significant variation across the area in the provision and quality of OG cancer surgery services had been acknowledged since the early 2000s.^16^ In addition, surgical services across GM had never achieved compliance with the standards in Improving Outcomes Guidance (IOG), as published in 2002^16^ due to catchment population sizes and surgical volumes undertaken. The need for the reconfiguration and centralisation of OG specialist cancer surgery services was generally recognised in GM but agreement about surgical sites had never been achieved,^16^ despite attempts by providers and commissioners to reconfigure services over the preceding decade and a half. These attempts failed for a variety of ‘informal’ reasons, for example recommendations were just ignored; and/or legal challenges to the process or outcomes. For example, one attempt was reported to the regulator for health services in England in 2012/2013 on a number of grounds including the role of providers in the decision-making processes (see Table 1). There had been some reduction in the number of hospitals undertaking OG cancer surgery out with formal service reconfiguration, from eight sites in the early 2000s to three at the outset of the reconfiguration attempt studied here (2015). The expectation was that this would be reduced to one site, based on clinical guidance for OG cancer.^16^

**Table 1:** Context, History, and Implementation: Reconfiguration of Oesophago-gastric Cancer Surgery Services in Greater Manchester

**Date**	**Governance and Leadership**	**OG Cancer Surgery Services**
2001	GMCCN established.	
2002	IOG published for OG cancer surgery services.	
2004	GMCCN clinical sub-group plans to meet IOG guidance submitted to the Department of Health – judged to be inadequate: Dr. Chris Harrison commissioned by SHA to review the plans.	
2005/2006	Association of Greater Manchester Primary Care Trusts established: formal decision-making authority to jointly commission health services across the area.	The Harrison Report: three specialist surgical centres recommended (to be implemented June 2007). No change implemented: recommendations *‘withered away.’*
2006-2009		Some specialist surgical centres ceased operating *‘out with formal commissioning processes,’* often linked to external peer reviews. Four non-compliant surgical centres remained.
2009	Commissioners requested review of OG surgical services.	The Alderson Review: two specialist surgical centres recommended. Decision challenged by ‘losing’ Trust on technical grounds, legal proceedings initiated, and procurement process halted. No change implemented.
2009		Two services in North Manchester combined voluntarily to create an IOG compliant centre: a total of three surgical centres remained (one compliant, two non-compliant).
2012	Manchester Cancer established: charged with working with non-compliant surgery services. Pathway Boards established. Greater Manchester Association of Clinical Commissioning Groups established: to lead CCG arrangements for specialised and joint commissioning; provide coordinated approach to service reconfiguration.	
2012/2013	NHS reorganisation – HSCA 2012.	Commissioner-led process to reduce surgical centres to two initiated. Referred to Monitor by two providers. Process abandoned.
2014/2015	NHS Five Year Forward review published (Oct 2014).	
2015	GM ‘took control of’ the £6 billion pa budget for health and social care: delegation of commissioning functions and resources to a joint commissioning board.	
07/2015	Briefing paper by the TU – see Table 2 – outlining the context for the future development of specialised services in GM and identifying OG cancer surgery services a priority for service transformation.	
	Transformation process for OG specialist cancer surgery services initiated.	
11/2015	GM Clinical Cancer summit held, providing an opportunity for clinicians, patients, carers, and providers to discuss the initial clinical standards which had been developed as part of the transformation process by the Pathway Boards.	
03/2016		OG Transformation workshop held by TU to ‘discuss the information and evidence base for potential service access requirements and engage with local experts to ensure that the service requirements are developed on the basis of local clinical knowledge and understanding of GM services.’
03/2016–06/2016		Five meetings of the OG External Advisory Panel held, to review and assure work being carried out planning the reconfigured services.
07/2016	Service specification endorsed by the GM Joint Commissioning Board.	Feedback meeting to OG Transformation workshop attendees, detailing model of care to be commissioned.
10/2016	Single site for specialist OG surgery announced by commissioners.	
03/2017		First meeting of OG Implementation Board.
05/2017–11/2017		Six meetings of OG Implementation Board.
01/2018	GMCA charge Chief executives of involved Trusts to implement OG service: OG Task and Finish Group chaired by Chief Operating Officer/Chief Officer to expedite the implementation.	
02/2018–08/2018		Six meetings of the OG Implementation Board.
09/2018		OG specialist cancer surgery service implementation: all specialist cancer surgery and benign complex surgery to be performed at the surgical Centre, GM-wide specialist OG on-call service operational.
12/2018	Official launch of GM OG specialist cancer surgery service.	

Abbreviations: SHA, strategic health authority; OG, oesophago-gastric; GMCCN, Greater Manchester and Cheshire Cancer Network; IOG, Improving Outcomes Guidance; CCG, Clinical Commissioning Group; NHS, National Health Service; HSCA, Health and Social Care Act; GM, Greater Manchester; TU, Transformation Unit; GMCA, Greater Manchester Combined Authority.

 In late 2015 another attempt to reconfigure OG cancer surgery services in GM commenced; in September 2018, the reconfiguration was completed. This process started before the formal devolution of the health and social care budget to GM, which commenced in April 2016. The reconfigured service consisted of a single surgical centre, a GM-wide specialist emergency on-call rota, three ‘sector’ multi-disciplinary teams and a Centre multi-disciplinary team.

###  Sample

 Non-participant observations (~160 hours) took place at meetings relevant for this analysis, including GM-wide cancer services governance meetings and meetings specifically focussed on the reconfiguration of OG cancer surgery services. In Table 2 there is a description of the boards and groups which oversaw the planning of the new services, and in the Supplementary file 1 there is information about additional relevant boards/groups at which observations were conducted. Table 3 displays the observations conducted. Observations were used as a data source in their own right as well as to complement and provide perspective on data collected in interviews.

**Table 2:** Boards/Groups Overseeing Planning of New Services

**Board/Group**	**Description**
GM Cancer	The integrated cancer system for GM and East Cheshire, bringing together the NHS services treating cancer with research into the disease and education of healthcare professionals.
TU	NHS Improvement and Strategic Transformation agency specializing inlarge-scale clinical services, healthcare consulting and change management. Services span the complete transformation cycle from developing a case for change through to implementation of new services or systems.
GMHSCP	Under a devolution deal with the government, the GMHSCP took over the health and social care budget for GM in April 2016.
Trafford CCG	Lead GM CCG for cancer (CCGscommission most of the hospital and community NHS services in the local areas for which they are responsible).
Greater Manchester Cancer Board	GM Cancer Board comprised cancer providers, commissioners, clinicians, people affected by cancer and other colleagues to reflect the entire cancer system. The aim was to secure world-class outcomes for the cancer patients and population of GM and East Cheshire.
External Clinical Assurance Panel	A panel of clinical experts (external to GM) who were asked to comment on and ‘assure’ clinical decisions such as clinical standards of care.
Specialised Commissioning	Leads commissioning for specialised services, such as OG cancer surgery.
OG Pathway Board	Board consisting of professionals involved in the care of patients with OG cancer, set up to oversee the whole OG cancer patient pathway and improve care for this group of patients.
OG Implementation Board	Set up to oversee the detailed design and implementation of the new specification and single site for OG cancer surgery in GM.
OG Clinical sub-group	Set up to oversee development of the clinical model and supporting pathways, operating procedures, and governance processes to meet the new service specification. Reported to the OG Implementation Board, providing updates and assurance at meetings, escalating issues where appropriate, and making recommendations to the Board.

Abbreviations: GM, Greater Manchester; TU, Transformation Unit; GMHSCP, Greater Manchester Health and Social Care Partnership; NHS, National Health Service; CCG, Clinical Commissioning Group; OG, oesophago-gastric.

**Table 3:** Observations Conducted

**Board/Event**	**No. of Occasions**	**Dates**	**Approximate Hours**
GM Cancer Board	14	February 2017–November 2018	28
Manchester Cancer Provider Board	6	Jan 2016–July 2016	12
GM Cancer summit	1	05/11/2015	6
Cancer Vanguard events	3	June 2016–September 2017	6
Cancer Vanguard Programme Board	1	June 2017	2
OG Pathway Board	15	May 2016–November 2018	30
OG Implementation Board	15	March 2017–August 2018	30
OG workshop and feedback meeting	2	March–July 2016	14
OG Clinical sub-group	7	August 2017–June 2018	10
OG Operational sub-group	14	August 2017–November 2018	21
OG staff information session	1	20/04/2018	1
**Total**	**79**		**160**

Abbreviations: GM, Greater Manchester; OG, oesophago-gastric.

 Documentary evidence (~300 documents) was gathered from online resources, meeting papers, and from stakeholders involved in the planning and implementation of the reconfiguration (Table 4).

**Table 4:** Types of Documentary Evidence Sourced

**Category of Document**	**Examples**
Background documents	Report from GM Cancer Summit 2012; OG cancer surgery service specification, OG cancer surgery commissioning specification; OG cancer surgery patient pathway.
Meeting minutes and accompanying papers	Obtained from MC Provider Board; GM Cancer Board; OG Pathway Board; OG Implementation Board; OG Clinical sub-group; OG Operational sub-group.
Documents relating to the process and progress of the reconfiguration	Case for Change document; GMCA and NHS GM project mandate; GMCA and NHS GM project initiation document; TU overview of planned process; briefings on progress and update letters.
Documents relating to specific events	Obtained from GM Cancer Summit 2015; OG staff engagement session; OG cancer surgery service launch event.

Abbreviations: GM, Greater Manchester; OG, oesophago-gastric; MC, Manchester Cancer; GMCA, Greater Manchester Combined Authority; TU, Transformation Unit; NHS, National Health Service.

 Interviewees were identified through documentary evidence, participation in meetings, and snowball sampling, and purposively sampled to reflect the range of different boards and groups which oversaw the planning of the new services; and the range of professionals who contributed to these boards and groups. Forty-six interviews were conducted (Table 5).

**Table 5:** Interviews Conducted

**Group/Board**^a^	**Designation/Role**	**Number of Interviewees**	**Number of Interviews**
GM Cancer	Director	3	6
	User Involvement	2	2
TU	Manager/Consultant	3	3
	Project Manager	4	6
	User involvement	1	1
GM Health and Social Care Partnership	Director	2	3
Lead CCG for cancer	Director	2	3
GM Cancer Board	Trust Director	2	2
External Clinical Assurance Panel	OG Consultant	1	1
Specialised Commissioning	Commissioner	1	1
OG Pathway Board	OG Consultant	3	3
	OG Nurse	1	1
	Dietitian	1	1
OG Implementation Board	Trust Directors	4	4
	Trust Project Manager	1	1
OG Clinical sub-group	OG Consultant/Oncologist	3	3
OG Operational sub-group	Trust Directorate Manager	4	4
	Trust Programme Manager	1	1
**Totals**		**39**^b^	**46**

Abbreviations: GM, Greater Manchester; OG, oesophago-gastric; TU, Transformation Unit; CCG, Clinical Commissioning Group. ^a^Interviewees are recorded against the board/group from which they were originally sampled. However, some interviewees belonged to more than one board/group. ^b^Some interviewees were interviewed twice.

###  Participant Recruitment and Data Generation

 Recruitment and data generation occurred between September 2015 and December 2018 and included meeting observations and individual interviews. Permission to observe meetings was obtained from the Chair. Meeting participants were supplied with participant information sheets and given the option to opt out of meeting observations: if participants were not happy, they could alert the Chair and the researcher would absent themselves for the whole or part of the meeting. Observations were recorded in the form of unstructured fieldnotes.

 Potential interviewees were approached via email with a participant information sheet attached. Interviews took place with fully informed written consent, the majority at the interviewees’ place of work. A semi-structured interview schedule was developed to reflect the different stages in planning and implementation of changes (Supplementary file 1). Interview questions defined the area to be explored but allowed interviewer or interviewee to diverge in order to follow up particular areas in more detail.^17,18^ Interviews lasted an average of 50 minutes, were digitally audio-recorded and professionally transcribed.

###  Analysis

 A thematic analysis^19^ of the interview transcripts, observation notes and documentary evidence was undertaken, initially using a deductive approach guided by the Best et al^1^ ‘simple rules’ framework and the work of Turner et al.^11^ As the importance of ‘attending to history’ emerged in early analysis, an inductive approach was used to explore how interviewees described this. Quotations are used to illustrate points made, labelled with participant number/professional role/meeting/document source.

 Further details about the data collection and analysis can be found in Supplementary file 1.

## Results

 Our findings develop two themes that have previously been identified as important in shaping how history is attended to: availability of personal and documentary historical accounts; and awareness of history.^6^ Ways in which history impacted on the work of planning and implementing the reconfiguration of OG cancer surgery services are analysed, with a focus on how those involved attended to history. The history of competition between provider sites was perceived as an issue to be addressed. Our findings also suggest a relationship between ‘attending to history’ and the other rules in the Best et al^1^ framework: designated and distributed leadership; engagement with stakeholders (including patients and families); and establishing feedback loops.

###  Availability of Historical Accounts and Awareness of History

 Documentary accounts of past attempts to reconfigure OG services in GM were available, for example reports of enquiries by the regulator in 2013, although there was no evidence in our data that interviewees had accessed these particularly. However, the proliferation of personal accounts of past attempts at change was very evident. This proliferation likely occurred because personnel, both managers and clinicians, tended to ‘stay’ in Manchester: “*GM doesn’t seem to have the churn [of personnel], perhaps, that other places I’ve worked at, and therefore their memories are long.*…*managers who are around for a long time” *(GM24/manager); *“People working in Manchester all their lives, you can do that with lots of hospitals in a small space…Manchester Medical School, Manchester Training Programme, Manchester consultant job” *(GM06/manager/clinician). Thus, there was a strong awareness of history among many interviewees, captured by the individual who, asked what the main challenge of the current reconfiguration process was, replied “*well history, I guess” *(GM03/manager).

 Differences were expressed about how in-depth awareness of history needed to be, possibly linked to an individual’s role in the planning of change. An interviewee with a strategic overview role thought that a broad understanding of history was more important than knowing the ‘nitty gritty’:

 “*Understanding some of the background is helpful....I understand the general background; when it comes to specific services, less so, and I’m not really hugely familiar with the history around OG, I don’t mean to be”* (GM31/manager).

 Other interviewees, with more of a ‘hands on’ role, considered that they needed to understand the detail of failed reconfiguration attempts. An individual who was involved early on in developing the proposal for change and planning the process commented:

 “*[It was] important to understand why previous procurement processes had not been successful and to do it differently basically … the initial stages were just examining the history, talking to people, understanding exactly what had got in the way of the procurement in the past. Then we designed an innovative process that took account of those failings” *(GM03/manager).

 Another interviewee, who served on one of the boards convened later in the process after the model of care had been agreed, talked about needing to know the intricacies of history: *“All the shenanigans that go on in meetings, the unsaid stuff, well if you don’t know anyone or any history, it’s quite hard to work that out” *(GM24/manager). This interviewee described contacting somebody after a meeting to ask why they had behaved in a particular manner, to be told a story about something that had happened in the past. We found no evidence to suggest that ‘history’ was explicitly contested by those we interviewed but the proliferation of personal, rather than documentary accounts, does imply that there may be several ‘versions’ of history depending on whose account is being drawn on and their particular perceptions.

###  History of Competition

 A history of competition between provider Trusts (organisations) in GM, for staff, reputation, and patients (therefore income), was perceived to have contributed to the previous lack of success in reconfiguring OG services. A number of factors were suggested to have engendered the competitive atmosphere: *“Lots of big Trusts, 3 teaching hospitals, all in a metropolitan area, all not wanting to give anything up” *(GM04/surgeon). The 2003 legislation enabling the establishment of Foundation Trusts, National Health Service (NHS) bodies with a degree of autonomy as decision making powers were devolved from central government to these local organisations, was also cited as influential: “*The seeking of Foundation Trust status put all the Trusts in active competition” *(GM13/surgeon). Although Trusts acquiring Foundation Trusts status was not unique to GM, interviewees perceived that GM was different to other areas because of the aforementioned lack of movement of personnel. This was thought to contribute to competitive thinking and loyalty to a single organisation, with staff *“very wedded to their organisation ”* (GM24/manager). It also contributed to the widespread knowledge of local competition amongst the interviewees.

 In response to the history of competition, those planning change put firm emphasis on the OG cancer surgery service as a GM-wide service involving all providers working together. This approach was evident from early on in the planning process, when attendees at workshops for local OG clinicians and managers in 2016 were encouraged to envisage a GM-wide service: *“We tried to create an environment of collaboration and cooperation”* (GM03/manager). As the process progressed, minutes from the first meeting of the OG Implementation Board, set up to oversee the detailed design and implementation of the new service, stated:

 “*Whilst the new service specification references a sole provider for [OG surgery], there will be a single service and it is important that organisations work together….all involved organisations should be proud of what they will achieve together” *(Minutes OG Implementation Board, 17/03/17).

 A manager on the Board commented:

 “*This is a GM-wide service….and that’s a slightly different way of thinking….compared to how things have run in the past. This is a single service for GM, it’s for the benefit of the entirety of GM; do you want to be part of the solution or part of the problem?” *(GM20(2)/manager).

 Although there was no specific reference by interviewees to the wider influence on the health system in GM at that time^20^ – devolution of health and social care funding and a GM-wide plan for the next five years (2016-2021)^21^ – it is plausible that the emphasis on a GM-wide service was at least linked to this (one interviewee suggested that reconfiguration of cancer surgery services represented *“an opportunity for an early devolution win”* (GM11/manager). Improving cancer care, and standardising acute and specialist care were two key priorities in this plan, which included the aim of ensuring that “highly specialised services requiring specialist skills and infrastructure will be organised at a GM level”^21^ (p. 39).

###  Influence of History on Other “Rules”

 The design of the change process was heavily informed by the history of challenges to previous reconfiguration attempts (see Table 1). This history was *“an undercurrent right through the programme ”* (GM01/manager) and ensured *“we’ve worked on the premise that the decisions will be challenged because they have been in the past” *(GM01/manager): the possibility of challenge *“informed everything we did”* (GM03/manager). The whole transformation process was designed to be collaborative, and to engage all relevant stakeholders from the start with the goal of avoiding challenge. To illustrate this, in the following sections we turn to how ‘attending to history’ influenced approaches to other rules in the Best et al^1^ framework by those planning and implementing change.

###  Designated and Distributed Leadership

 Designated leaders are formally in charge of a programme of work, distributed leaders are people and teams who share responsibility for implementing a programme and its components: all of these people are change leaders.^1^ We focus here on designated leadership, the context for which is that since 2013, specialised services for OG cancer surgery have been commissioned by NHS England. In 2016, health and social care funding was devolved to GM, and the Greater Manchester Health and Social Care Partnership (GMHSCP) established. Alongside the devolution agreement, a memorandum of understanding between GMHSCP and NHS England included the commissioning of specialised services. The GMHSCP therefore had the role of designated leader and commissioned and funded an NHS consultancy (NHS Transformation Unit: TU – see Table 2) based in GM, and with experience of working there, to facilitate the creation of a single service model for specialised OG cancer surgery services and support its implementation. Whilst governance arrangements were enabled by the devolution process from April 2016, it was the history of failed attempts to reconfigure services led purely by providers or commissioners which influenced the development of a different leadership structure:

 “*….using [the TU] to almost be an independent, so neither a provider or a commissioner but with expertise in transformation, to come up with something that both providers and commissioners could sign up to” *(GM10/manager).

 The TU designed an eight-step transformation process involving service providers, patients and the public putting together clinical standards, after which the TU worked with providers to develop service access frameworks and explore potential models of care. From this, the TU designed the final model of care and service specification, acting as facilitators of the various groups and boards in the process, allowing commissioners to then make decisions about where services should be provided.

 The TU was keen to emphasise that the process was engaging with provider sites appropriately, as previous change attempts had been halted in part because of fear from a range of stakeholders (expressed in personal accounts of history) that providers had too much influence on the placement of services. Having engaged with providers through the OG Pathway Board (a multi-professional group charged with overseeing the OG patient pathway from a clinical perspective) about clinical standards for the OG service, the TU wrote to the Board thanking them and saying that the TU would take the clinical standards and use them to develop the service specification. A member of the Pathway Board, representing providers, commented that this occurred so that *“I could not contaminate the process [of developing the service specification]”*(GM10/manager).

 The independent role of the TU was viewed positively by interviewees:

 “*They’ve [acted] in a very independent honest broker kind of way. They administer the meetings, support…the implementation group. And where there’s organisational difficulties they support the Board in overcoming those. Where you need exec level involvement, they can do that” *(GM10/manager).

###  Engagement With Stakeholders

 Whilst the ‘simple rules’^1^ refer mainly to engagement with physicians, subsequent studies ^11,22^ have indicated engagement with a wide range of stakeholders is crucial. Change leaders in GM (distributed across the system) understood that the history of attempts to change had affected how stakeholders viewed, and engaged with, the current attempt; an example of history as a barrier to change. One manager commented that “*a process that’s failed so many times…. there is an issue associated with credibility; why should we embark on this again when patently the last time it failed?” *(GM16/manager)and another spoke of stakeholders asking,* “what makes this different, why do you think it’s going to make any change?”* (GM12/manager). By maintaining awareness of these attitudes, change leaders were able to work to encourage engagement.

 This was achieved partly through acknowledging past experience: *“I stood up at the front quite purposely and said, ‘We recognise you’ve been marched up the hill, but we’re making a personal commitment [to change]’” *(GM11/manager), and partly through designing a change process *“that was so obviously different that it didn’t look like trying to do a failed process”* (GM16/manager). In addition, change leaders talked about working individually with stakeholders who were reluctant to engage, *“lots of one to ones”* (GM11/manager): *“Lead clinicians… had to be persuaded, conversations took place to persuade people that we need to make change and this time we are actually going to do it” *(GM12/manager).

 Understanding the history of the relationships between stakeholders was important in engaging stakeholders effectively. A manager commented: *“One of the reasons that these services have never been sorted is that surgeons are big personalities, that’s how they’re bred….they have a lot of history, they’ve known each other for a long time, they’ve trained each other”* (GM11/manager). These relationships were taken into account, for example a Manager spoke about the seating plan at an engagement event:

 “*… if you have certain personalities together, they are not going to come up with a consensus decision because whatever one says somebody will say the opposite… the way [the TU] stage-managed the tables around who was sat where was helpful around gaining consensus” *(GM12/manager).

 Change leaders were particularly keen to encourage wide stakeholder engagement as they perceived that past change attempts had failed because engagement and participation in developing plans for change had been absent. One way in which this was understood to block change was because of a lack of ownership of change. Referencing the 2005-2007 attempt to reconfigure OG services (Table 1) a surgeon commented:

 “*There was never a decision that said we’re going to stop this process…. everyone just ignored it….nobody did anything and the thing just withered away”* (GM06/surgeon).

 Lack of early engagement was also understood to result in active opposition to change. A manager spoke about his/her experience of this in relation to previous attempts to reconfigure OG services:

 “*We needed to come up with something that was different enough to... designing something, putting it to clinicians and providers and then finding that somebody, if they are not comfortable with the outcome perhaps, might find another systemic failure in the process [and consequently challenge it]” *(GM16/manager).

 These experiences led to a deliberately inclusive approach, with the belief that if commissioners, service providers and users worked together from the start, change would happen. A surgeon stated, “*there’s been every attempt made to keep this open [with] clinician involvement from the outset, service user involvement from the outset”* (GM06/surgeon).A manager representing commissioners described using principles of *“co-design and getting ownership and buy-in to an overarching process” *(GM16/manager). Another explained:

 “*….working collaboratively with the providers who have an understanding first-hand of the issues of running services day-to-day. You tap into their knowledge and expertise, that was very important, just that collaboration and cooperation and engaging with the clinicians from the start” *(GM03/manager).

###  Establish Feedback Loops

 The Best et al^1^ rule establish feedback loops referred to identifying and feeding back to those involved in MSC outcome measures, to demonstrate the effectiveness of change. In this GM OG reconfiguration, feedback loops in relation to the process of planning the reconfiguration were established, since outcomes were not expected to change until the reconfiguration had been done This was in response to the challenges that previous reconfiguration attempts had received on both clinical and procedural fronts, which change leaders were very aware of. External feedback loops were used from the outset to assure both clinical decisions (such as the clinical standards), and the transformation process itself. Change leaders talked about ‘clinical assurance’ and ‘process assurance.’

 In terms of clinical assurance, an External Clinical Assurance Panel was set up at the beginning of the process, consisting of clinical experts from outside GM, along with patient representatives. This approach had not been used before and was designed to address challenges to the changes on clinical grounds. The group examined and commented upon the outputs of every step of the planning process:

 “*[They] played an important role in terms of providing that external assurance…very helpful in terms of challenging us on the standards and then, in terms of the service access framework as well, they were very insightful, and helpful” *(GM03/manager).

 In terms of process assurance, those planning the reconfigurations ensured that relevant regulatory bodies were informed about the work that was being undertaken. For example, previous reconfiguration attempts had been referred to Monitor (the regulator at the time), so change leaders ensured awareness of the current reconfiguration plans and invited feedback on the process:

 “*We also involved Monitor very early on and invited them to events. They didn’t always turn up, but they got all the paperwork and we maintained regular discussion with them…although they don’t give advice, we did share our process as it was developing with them and ask for any comments they had….to make sure that the potential for challenge was minimised ” *(GM03/manager).

 In addition, change leaders talked about feeding back about progress to senior stakeholders in provider trusts at regular intervals throughout, in order that challenges to the reconfiguration could not be made on the basis that individuals or institutions had not been informed about what was happening, as had occurred previously:

 “*We regularly met with the Chief Executives so that they were very well aware of what was going on, there were no surprises, and we did regular updates. I used to write a briefing, just a couple of sides of A4 every 4-6 weeks just to keep everybody up to speed with what was going on, again with the aim of minimising challenge”* (GM03/manager).

 The whole ‘eight-step’ transformation process was designed so that feedback was received at each stage and the decisions that were made were ‘locked down’ in order that they could not be revoked:

 “*It’s kind of a step-wise iteration process so there’s various points along the pathway where the commissioners lock down what’s been agreed already, the idea being that you can’t go back hopefully and question the process, the nuts and bolts, the logistics, the administration of the process, as has happened before”* (GM06/surgeon).

## Discussion

 It has been argued that attending to history is important in MSC, but there is relatively little empirical evidence to support this^1^ and there has been little unpacking of what the term means or the ways in which it may be interpreted. Through attending to history in terms of both personal and documentary accounts, those leading the reconfiguration of OG cancer surgery services in GM developed a change process which took into account previous unsuccessful reconfiguration attempts, enabling them to reduce the impact of potentially challenging issues. It was recognised that having a change process within the context of competition; led by any one single stakeholder group (commissioners or providers in isolation); with poor stakeholder engagement; and processes amenable to challenge contributed to the failure of previous reconfiguration attempts (Table 1). The change process analysed here took these issues into account and utilised an independent broker for the process (the TU), who maintained an awareness of how previous change attempts had affected the willingness of local stakeholders to engage in change. Although the TU did not explicitly describe their approach as ‘attending to history,’ it is clear that history was taken into account in the process described by our interviewees.

 Using Suddaby and Foster’s^4^ perspectives on history is a useful way of exploring the ways in which history was attended to. Two of the perspectives were particularly evident in our data: History-as-Power and History-as-Sensemaking.

###  History-as-Power

 Taking a History-as-Power perspective^4^ highlights that the focal point of change was not organisational design but the power structure of the various stakeholder coalitions, and the power differences which are ‘solidified’ through history, but also acknowledges the ability of individuals to reflect upon the history of power relations and act on them. Given the proliferation of historical accounts, and the awareness of history described in the findings, it was possible to identify clearly how change was “*characterised by long periods of relative inertia maintained by countervailing political pressures*”^4^ (p. 26) and that change finally occurred when a different approach was taken. The History-as-Power perspective enables insight into how lack of professional agreement was a stumbling block to previous plans, influenced by the competitive context. This seemed to be the view of those at GM level attempting to progress this reconfiguration although not expressed explicitly as such by our interviewees. The aim appeared to be to get an agreement rather than to push a specific model of reconfiguration, with the TU facilitating that process as a neutral broker, with no ‘interest’ in pushing a specific model although putting emphasis on a single GM-wide service rather than focusing on individual organisations. Although some stakeholders from individual organisations clearly preferred some options over others, the focus was more on assuring the process and making it resistant to challenge from any specific stakeholder group – clinical or otherwise.

 Another example of reflecting on historical power relations can be seen in the way in which professional (medical) power was handled. Some of the issues encountered during the change process, both past failed attempts and the one studied, were related to professional power, as noted by various authors including Addicott and Ferlie,^23^ who also studied cancer services. The stage managing of events to keep big personalities apart, which was something developed as a result of knowledge of previous change attempts (history) could be regarded as exercising of top down managerial power. It could also be viewed, however, as a pragmatic approach to move towards a decision, facilitated by those independent of (although commissioned and funded by) senior system managers. This History-as-Power perspective provides insight by suggesting that it enables the overcoming of “the constraints of history through retrospection, critical reflection and creative visioning”^4^ (p. 26) and was arguably the approach taken and enabled by the TU.

 During the development of the service specification and model for the OG cancer surgery service, professional power was deliberately limited to one particular stage of the process – until clinical standards had been agreed (see Table 1). This separation of clinical involvement and the ‘service specification and model’ process could be viewed as an attempt to limit the impact of professional power, which had contributed to the failure of previous change attempts. The one-to-one conversations between the TU and various key stakeholders was also evidence that professional power was recognised, and attempts were being made to mitigate its negative impact on the process.

 The availability of both personal and documentary historical accounts is important when attending to history^1^ and it might be that personal accounts lead to a History-as-Power perspective whereas documentary accounts are less contested and are viewed from a History-as-Fact perspective. In this study there was a wide awareness of the history of change attempts in OG cancer surgery services in terms of both power and facts. It may be that this was particularly acute because of the plethora of personal historical accounts that were evident: almost everybody could ‘tell a story’ about previous change attempts, whether they had been involved themselves or not, although these always represented their personal interpretation of events. This ease of access to information perhaps contributed to an atmosphere where it was difficult *not* to attend to history. It may be that in situations where historical accounts are mainly documentary (History-as-Fact) and not a topic of discussion, there is less likelihood that history will be so widely known or to such a level of detail, and hence acted upon. In this study the extent of discussion about history led to consideration of power in the process of change described here.

###  History-as-Sensemaking

 The ‘History-as-Sensemaking’ perspective can also be identified in the approach of change leaders. This perspective holds that organisational reality is based on how participants interpret their collective experience and privileges the human interpretation of events over “brute facts.”^4^ (p. 27). The approach to change studied here recognised that a history of failed attempts affected how stakeholders perceived the current process and how willing they were initially to engage with the planning of the changes. Thus, the wide stakeholder consultation and the willingness of the TU to acknowledge stakeholders’ past experiences seemingly contributed to the successful planning and implementation of the OG cancer surgery service. It could be argued that the use of the TU as an external agent to enable the change process to move forward, is an example of the manipulation of stakeholder consultation to serve ‘powerful interests,’ as described by Fraser et al.^24^ These authors described the way in which management consultants were able to control how problems were understood, and which solutions were adopted, which can be perceived as similar to the way in which the TU developed and implemented their eight-step process. However, we identified positive views from stakeholders about this process and the independence of the TU, which might be explained by the fact that the TU had acknowledged stakeholders’ previous experience and made stakeholders more positively inclined to this different ‘independent broker’ way of working.

 The evidence here is not consistent with the view that stakeholder engagement is a co-optive device,^25^ where involvement is recognised as such by stakeholders and can then backfire by eroding trust. Given the wide range of stakeholders interviewed in this study and the time period over which the data were gathered, it is likely that such co-option would have been identified. Instead this study demonstrates that “trust rather than simply empirical evidence… is key to the acceptance of change in intractable controversies”^25^ (p. 202). Stakeholders were being asked (albeit indirectly) to trust the process, not to trust any one party or group and it is notable that we found little debate about the empirical evidence, compared to the process as described in the historical accounts.

###  Interaction of Attend to History With the Other Simple Rules

 Drawing on Suddaby and Foster,^4^ the Best et al^1^ ‘attend to history’ rule arguably suggests a History-as-Fact “*objective, positivist view of history*”^4^ (p. 22), and therefore conceptualises change as difficult, focused at organisational level and resulting in new structures or operations. Through the example of reconfiguration of OG cancer surgery services in GM, we have demonstrated that attending to history is broader than History-as-Fact.

 The study highlights how history intersects with the other rules in the Best framework.^1^ Through ‘attending to history’ and viewing history not only as-Fact, this study shows that change leaders also influenced other rules, in this case, aspects of leadership, stakeholder engagement (previously described) and feedback loops, although they may not have been explicit about this.

 History informed the approach to leadership, both within the system and through the ‘independent’ role of the TU itself. The use of formal feedback loops, with an emphasis on external assurance as well as a clearly articulated change process, was a result of the history of the previous failed change attempts. Through the recognition of history both as power and sensemaking, in addition to History-as-Fact, history may influence the other rules as this study has shown. Historically informed leadership arrangements and stakeholder engagement, led to an approach more nuanced to the context within which it was being undertaken, and one which attempted to take into account the power of the various groups involved ie, recognising subtleties and inter-stakeholder dynamics.

###  Limitations of the Simple Rules Framework

 The broader perspective offered by disciplines such as sociology enables insight into how the findings of this study show the limitations of the simple rules framework. A previous critique of the Best et al^1^ simple rules states that a more sociological perspective might ask:

 “*How does the use of ‘simple rules’ frame phenomena or influence the reader? Why is large-scale change assumed to be a good thing? … Why are issues of politics and power excluded from analysis or reduced to formal institutions?”*^26^ (p. 1224).

 Framing of an issue, and the findings from research, has been shown to be important and potentially influential in the outcome of a process of change.^25^ In their study of hospital planning, Jones and Exworthy^25^ stated that “*the framing disguised the political nature of the issue by defining it as a clinical problem*” (p. 196). In our study the accounts of history demonstrated the complexity, and political nature, of the changes being planned. Whilst clinical issues were regularly cited and subjected to external assurance through the Clinical Reference Group, framing as solely a ‘clinical problem’ did not occur as it had in previous change attempts. Instead the issue was framed as a ‘process’ issue, potentially disguising the political nature of the change. If framing is defined as channelling thinking and making a particular course of action appear self-evident,^25^ then in this study the channelling was to a series of steps/process with a decision being self-evident, rather than the specific outcome of that decision being the focus of the channelling. Whilst it is highly likely that there were stakeholders who wanted one specific outcome/configuration of services, reframing the issue as one of process rather than outcome appears in to have led to action (ie, reconfiguration), which had previously not been achieved.

 Framing can also be viewed as a form of discursive power:

 “*…discursive power, which through an emphasis on evidence, better patient outcomes, professional support and clinical credibility alongside a tightly managed consultation process, helped to set an agenda that was broadly receptive to the overall decision to change … services”*^24^ (p. 1).

 There are parallels here with the study by Fraser et al^24^ of stroke services reconfiguration, where evidence-based medicine was used as a “*technique of power … through which problems are constructed and understood*”^24^ (p. 7) and also in a wider study^27^ that included the stroke case which led, as in our study, to a decision to change services. Discursive power was clearly demonstrated in our study with the need to change not really questioned, but rather the extent and scope of the change required being the sticking point (see next section). There was a tightly managed process, not only in terms of consultation but also in terms of planning and implementation, to attempt to ensure that change actually happened.

 Change is more complex than a solution being imposed by managers against the wills of clinicians, as this study, and many others, show, so perhaps the use of the term ‘simple rules’ is itself misleading. Within the context of an area like GM (population ~2.8 m), the reconfiguration of a single cancer service was considered by the policy-makers (in this research, GMHSCP) to be good and part of their wider strategy for GM. The change also had wider implications for other health services especially where hospitals who did not provide the cancer surgery service after reconfiguration might then see impact on their capacity to staff other non-cancer clinical rotas. The GM level plan,^21^ and the clinical case for change, described both patient benefit and also efficiency savings/financial sustainability, which was itself a key element of the rationale for devolution.^28^ As such, change might not have been viewed by all as a good thing but was the implication (and arguably one of the objectives) of the GM health and care system at that time.

 This study shows that the use of a series of ‘rules,’ without consideration of their interaction and the subtle power dynamics between stakeholders and over time, provides only a limited perspective on the issues of system change. We argue that the simple rules framing can be useful - but we demonstrate here its limitations, the interaction between the ‘rules’ and in particular the influence of ‘attending to history’ on all other aspects has been shown in this study to be crucial in achieving the outcome identified by the health system as desirable. The paper extends existing knowledge about how history may influence other aspects of change processes and the importance of stakeholder power within that.

## Conclusion

 Whilst there were changes in the context of the reconfiguration studied here, in terms of the devolution of funding to GM and other influences which arguably encouraged the development of pan-GM services, our data indicate that these changes would not alone have been enough to ensure reconfiguration, given the competitive nature of the system and the history of previous change attempts. It was the combination of the context becoming arguably more supportive of reconfiguration, along with the recognition of, and response to, history from more than one perspective, which led to the OG surgery changes being successfully implemented ie, reconfiguration taking place.

 The claim that “*History offers a valuable but underexplored organisational resource that can be useful to motivate and successfully manage change*”^4^ (p. 35) is borne out by this study. Linking this with the ‘simple rules’ (although this study might suggest that they are in reality far from simple) by considering history from a range of perspectives – in particular, history as ‘fact’ as well as ‘power’ and ‘sensemaking’ – and recognising its interaction with the other ‘rules’ enables powerful insight into system change which can be of use in future changes. The paper extends existing knowledge about how history may influence other aspects of change processes and the importance of stakeholder power within that. Change leaders might do well to be aware of both documented history and accounts of history from key stakeholders, as well as considering their power within the system, when designing and implementing change processes.

## Acknowledgements

 The authors acknowledge the contribution made to this study by Neil Cameron, one of the patient representatives on the team, who died in May 2017. They also thank all of the members of the RESPECT-21 Research Strategy Group for their contributions to the development of this paper. Finally the authors would like to thank all those people who generously gave their time to participate in this research.

## Ethical issues

 This study received ethical approval in July 2015 from the Proportionate Review Sub-committee of the NRES Committee Yorkshire & the Humber–Leeds (Reference 15/YH/0359).

## Competing interests

 Authors declare that they have no competing interests.

## Authors’ contributions

 NJF, AIGR, RJB, and DS made substantial contributions to the conception and design of the work. CP, SD, GBB, CVP, AIGR, and CSC contributed to the acquisition and analysis of the data for the work. All authors made substantial contributions to the interpretation of data for the work. CP and RJB drafted the work and all other authors revised it critically for important intellectual content. All authors gave final approval of the version to be published. All authors agree to be accountable for all aspects of the work in ensuring that questions related to the accuracy or integrity of any part of the work are appropriately investigated and resolved.

## Funding

 This study presents independent research commissioned by the National Institute for Health Research (NIHR) Health Services and Delivery Research Programme, funded by the Department of Health (study reference 14/46/19). NJF is an NIHR Senior Investigator. The views expressed are those of the authors and not necessarily those of the NHS, the NIHR or the Department of Health and Social Care. GBB is supported by the Health Foundation’s grant to the University of Cambridge for The Healthcare Improvement Studies Institute.

## Supplementary files

Supplementary file 1. Further Details About Data Collection and Analysis.
