# Supplementary material for: "Attending to History" in Major System Change in Healthcare in England: Specialist Cancer Surgery Service Reconfiguration
Source: Int J Health Policy Manag. 2022 Mar 14;11(12):2829–41. doi: 10.34172/ijhpm.2022.6389 (PMC10105206; doi:10.34172/ijhpm.2022.6389)
Supplement: Supplementary file 1 — Further Details About Data Collection and Analysis. [file ijhpm-11-2829-s001.pdf]

Article title: “Attending to History” in Major System Change in Healthcare in England: Specialist Cancer Surgery Service Reconfiguration

Journal name: International Journal of Health Policy and Management (IJHPM)

Authors' information: Catherine Perry[1,2]*, Ruth J. Boaden[3], Georgia B. Black[4], Caroline S. Clarke[5], Sarah Darley[6], Angus I.G. Ramsay[4], David C. Shackley[7,8], Cecilia Vindrola-Padros[9], Naomi J. Fulop[4]

[1]Applied Research Collaboration Greater Manchester, Division of Population Health, Health Services Research and Primary Care, University of Manchester, Manchester, UK.

[2]Division of Nursing, Midwifery & Social Work, School of Health Sciences, Faculty of Biology, Medicine & Health, University of Manchester, Manchester, UK.

[3]Alliance Manchester Business School, University of Manchester, Manchester, UK.

[4]Department of Applied Health Research, University College London (UCL), London, UK.

[5]UCL Research Department of Primary Care and Population Health, University College London (UCL), London, UK.

[6]Centre for Primary Care and Health Services Research, University of Manchester, Manchester, UK.

[7]Christie NHS Foundation Trust, Manchester, UK.

[8]Institute of Cancer Sciences, Manchester Academic Health Science Centre (MAHSC), Manchester, UK.

[9]Department of Targeted Intervention, University College London (UCL), London, UK.

(*Corresponding author: Email: Catherine.perry@manchester.ac.uk)

Supplementary file 1. Further Details About Data Collection and Analysis

Additional Boards/Groups at which observations were conducted

Board/Group	Description
Manchester Cancer Provider Board	Oversaw the work of Manchester Cancer, the predecessor to Greater Manchester Cancer.
GM Cancer Summit	The purpose of the Summit was to discuss, refine and ratify, with the wider cancer community, the initial draft of the patient experience and clinical standards for Urology and oesophago-gastric (OG) Cancer which had been developed by patients and Urology and OG clinicians.
Cancer Vanguard Programme Board	Oversaw the work of the GM Cancer Vanguard, one of three partners in the national Cancer Vanguard, which was set up to pilot new models of cancer care.
Cancer Vanguard Events	Public events held to showcase the work of the GM Cancer Vanguard.
OG workshop	Workshops held by the Transformation Unit for local health professionals and managers working in OG cancer care which aimed to agree the vision for the future OG cancer service in GM.
OG staff information session	Session for staff affected by the OG service changes, aiming to explain the impact of changes and answer questions staff had.

Interview Schedule

Note: This is a summary of general topics: some will be of limited or no relevance to certain of our interviewees.

1 General introduction

- What is your current role/post and what does it involve?
- How are/were you involved with the reconfiguration of cancer surgery services?
  - How are/were you involved with the reconfiguration of individual pathways?

2 Background to the reconfiguration

Can you tell me about the proposals to reconfigure specialist cancer surgery?

- How did you first hear about these proposals? What did you think? And your colleagues?
- What were the catalysts or drivers for the reconfigurations (OG and/or Urology)? How was the decision made? Why did people decide to change?
  - *national policy? local drivers/factors? key players?*
- Who was consulted on these changes?
- Was a lot of time/resources spent at this stage? Extra staff?
- Can you tell me about any prior attempts to try to reconfigure specialist cancer surgery/why didn't change happen before?
- What do you think were the main challenges at the beginning of the current process?

3 Developing the proposal for change/agreeing the reconfiguration model

Can you tell me about how the new models of care were developed and agreed?

**Components of change**	**General prompts**
Case for change<br>Clinical standards<br>Patient experience standards<br>Patient and clinical engagement and involvement<br>Service access frameworks<br>Model of care<br>Service specifications and patient pathways<br>Specialist/local site recommendations	How were you involved? Time dedicated? Time dedicated by support staff?<br>Obstacles/enablers/challenges<br>What did you/your colleagues think?<br>Assurance – process, clinical, scrutiny

4 Implementing the model

NB May need to go through this set of questions separately for OG and Urology.

Can you tell me what the position is at the moment (for OG/ for Urology)?

- What are the overall timeline for the reconfigurations?
  - *Were there any factors that drove the timelines (OG and Urology)? Amendments to timelines?*

Can you tell me about how the changes are being/will be implemented?

- What kinds of changes will have to happen to enable implementation?
  - *In refocusing services, changes in activity, changes to contracts (joint), job plans, building capacity, leadership training, changes in referral pathways, ensuring new governance processes.*
  - *Phased approach.*
- How are/will you be involved in implementing the changes?
- Which groups and individuals are/will be central to implementation? How do they/will they work?
  - *Prompts: The OG (Urology) Implementation Board: how was the Board set up, what is the remit of the Board, how does it fit into existing governance structures, what is your role on the Board, is the membership of the Board right, what did you initially see as the major challenges, have those changed, what about enablers?*
  - *Initial work of Board: OG - three 'quick wins' (elective surgery from UHSM/CMFT, pan GM SMDT, emergency rota), aspirational interviews. Urology – prostate surgery, SMDT.*
  - *Prompts: sub-groups (role, membership, working),*
  - *Prompts: any specific issues that have arisen (signing of MOU)?*
- Training? (Information days). Where/how many?
- Obstacles and enablers-how are/will these be addressed? What are/will be the levers for change?
- How long might implementation take and why?
- Are there/might there be any implementation costs or resource requirements that were not anticipated during the planning stages of the reconfigurations?
- How are/will local stakeholders be kept up to date on progress of the reconfigurations?
- How will patients/the public be kept up to date on progress of the reconfigurations?

5 Governing the reconfiguration

Can you tell me how the reconfigurations were/are organized and governed?

- Which groups and individuals lead and govern the reconfigurations? Any new staff appointed/seconded specifically (detail)?
- What are the roles and responsibilities of these groups and individuals?
  - *Prompt: GM Cancer, commissioners,* ***patient groups,*** *pathway leads.*
- How are you involved?
- What are some of the key meetings and events?
  - How do these meetings and events work? How frequent are they?
  - What are some of the challenges encountered at these meetings and events?
- What were the resources allocated for the reconfigurations?
  - *Prompt: funding for Manchester Cancer/Greater Manchester Cancer, other types of funding, staff.*
  - *Was this funding enough? Too much?*
  - *Did additional funding need to be obtained? Where from? How much?*

6 Outcomes

What changes do you anticipate will be brought about by the reconfigurations?

  - *Prompts: organisation, service delivery, partnership working, patient outcomes, costs, patient and carer experience (choice and continuity of care, problems such as issues with travel), staff experience (emphasise ways of working, skill mix and approaches to collaboration)*

- What are the outcomes you expect from the reconfigurations?
- How will they be measured? What capacity is/will be dedicated to collecting these data? Are these measures reliable?
- What factors may influence sustainability of changes and what is being done to facilitate this?
- Would these changes have happened anyway?
- Do you feel the resources put into the design and implementation of the reconfigurations will have been worthwhile in the short term? Long term? Are you recording any information about money spent on planning and implementation stages?
- Are there any negative outcomes/impacts? Or unanticipated outcomes/impacts?

7 Reflections

What lessons have you drawn from this? Is there anything you would have done differently?

- If other areas in the country were to undergo this type of reconfiguration, what advice would you give them? (*NB commissioners might wish to reflect on different healthcare domains*)
- Is there anything you think we should know that I have not asked you?
- Have you any further comments?

Thanks for time and contribution.

Further information about qualitative data collection and analysis

Interviews were conducted by one researcher (CP) and observations were carried out by two of the research team (CP/SD). Both researchers were female, held a PhD, and were experienced in the research methods used. There was no relationship between research participants and researchers prior to the commencement of the study. The researchers gave verbal presentations to many of the boards/groups attended, explaining their role as University researchers. Similar explanations were given to individual interviewees. Due to the longitudinal nature of the study, with data being collected over a period of three years, the researchers and research participants got to know each other on a professional basis. Familiarity with the researchers by some research participants may have contributed to the richness of the data collected.

None of the groups/boards approached for the purpose of observation declined to take part. The majority of potential participants who engaged with the researchers were willing to be interviewed, with <5 people not responding to an email invitation. The interview schedule was developed by the researchers, overseen by the project Research Management Group, which included University researchers, NHS clinicians and managers. It was reviewed over the course of the interviews to ensure that relevant data were being generated. Interview participants were offered sight of transcripts, although only a very small minority availed themselves of this. One interviewee did not agree to be audiorecoded (due to concerns about freedom of information requests).

Interview transcripts were uploaded onto NVivo software to aid data management. Steps were taken to enhance methodological rigour. The initial analysis was carried out by one researcher (CP) and the emerging analysis was shared and discussed with a small subgroup of the authors (RB/GB/SD/AR/CV). The emerging analysis was also discussed with the project Research Management Group (to which all of the authors belonged) and the Research Strategy Group, which included all of the project collaborators, including clinical leaders of London Cancer and Greater Manchester Cancer, the relevant pathway leads, and patient representatives. Analysis and findings were further discussed with the Study Steering Committee which included a wide range of national clinical and patient stakeholders.
